# Retrograde signals galore

**DOI:** 10.3389/fpls.2013.00045

**Published:** 2013-03-12

**Authors:** Tatjana Kleine, Dario Leister

**Affiliations:** Plant Molecular Biology (Botany), Department Biology I, Ludwig Maximilians UniversityMunich, Germany

The term “retrograde signaling” refers to the concept that signals originating from chloroplasts or mitochondria can modulate nuclear gene expression (NGE). The evolutionary establishment of the complex mechanisms underlying retrograde signaling is likely to have required a long time period (reviewed in: Tanaka and Hanaoka, 2012). The initial notion, proposed almost 30 years ago, that a single plastid signal might regulate the expression of nuclear genes involved in plastid biogenesis (Oelmüller and Mohr, 1986) has since expanded to accommodate the insight that multiple signals are produced by plastids (Gray et al., 2003). While the ultimate effects of retrograde signaling on NGE have now been clearly defined, many aspects of the initiation and transmission of the signals, and their mode of action, remain unresolved, are a matter of speculation (Estavillo et al., 2012), or are controversial (Kleine et al., 2009). Relevant signals are thought to derive from various sources, including (1) reactive oxygen species (ROS), (2) the reduction/oxidation (redox) state of the organelle, (3) organellar gene expression (OGE), and (4) the tetrapyrrole pathway, and in the present volume Estavillo et al. (2012) describe “brand-new” retrograde signaling pathways involving (5) metabolites—particularly 3′-phosphoadenosine 5′-phosphate (PAP) and methylerythritol cyclodiphosphate (MEcPP)—and (6) a carotenoid derivative (β-cyclocitral [β-CC]).

The collection of Original Research (all conducted with the model plant *Arabidopsis thaliana*), Hypothesis and Theory, Mini Review, Review, and Perspective articles assembled for this Research Topic reflects not only the progress made in characterizing the four “classical” signal sources, but also highlights alternative interpretations of how information transfer from chloroplasts and mitochondria might be mediated and how the activities of these two organelles might be coordinated with that of the nucleus. Taken together, the contributions suggest a framework for future research in the retrograde signaling field.

## Progress in ROS-associated signaling

Photosynthetic organisms must continuously cope with ROS, such as singlet oxygen (^1^O_2_), the superoxide anion radical (O^2−^), the hydroxyl radical (OH^·^), and hydrogen peroxide (H_2_O_2_), more especially when exposed to stresses (Apel and Hirt, 2004). Shapiguzov et al. (2012) summarize evidence for the influence of extracellular and chloroplastic ROS production on NGE and describe the routes by which such signals might reach the nucleus. However, the extent to which the precise chemical nature of ROS and their cellular compartment of origin may contribute to the multiplicity of responses that occur in plants is still unknown. Vestergaard et al. (2012) model intracellular H_2_O_2_ signaling via diffusion using a H_2_O_2_ signal originating at the plasma membrane and spreading through the plant cytosol. These authors conclude that, although diffusion-mediated signaling is theoretically possible, it is unlikely to work in practice, since it requires a much faster rate of enzymatic degradation and a much lower cellular background concentration of H_2_O_2_ than are observed experimentally. Plants overexpressing peroxisomal glycolate oxidase (GO) in plastids (GO plants) can be exploited to study the effects of plastid-generated H_2_O_2_ (Fahnenstich et al., 2008). In GO plants, genes that respond strongly to an induced, abrupt rise in levels of plastid-generated H_2_O_2_ encode proteins involved in the regulation of anthocyanin biosynthesis, as well as transcription factors and their interacting partners that affect development (Balazadeh et al., 2012).

Partial exposure of low light-adapted plants to excess light results in systemic acclimation to excess excitation energy and the attendant photo-oxidative stress in unexposed leaves (Karpinski et al., 1999). Gordon et al. (2012) show that transient changes in light intensity imprint a “memory” of the event that facilitates subsequent acclimation responses. Acclimation to high light (HL) is induced within minutes, and repeated exposure to short-term HL results in acclimation of the exposed tissue and that of emerging and young leaves (but not older leaves) to HL and oxidative stress. The double mutant *adg1-1 tpt-2*, which is defective in the small subunit of the ADP-glucose pyrophosphorylase (ADGase; a key enzyme in the starch biosynthetic pathway) and the triose phosphate/phosphate translocator (TPT; the major interface for the distribution of photoassimilates between the chloroplast and the cytosol), shows impaired acclimation to HL, resulting in impairments of the diurnal pattern of photoassimilate export from the chloroplast (Heinrichs et al., 2012). The rescue of this double mutant by exogenously supplied sugars provides evidence that chloroplasts are capable of directly sensing the carbohydrate status, and it raises the question whether retrograde signaling is actually necessary for sugar-mediated acclimation processes (Heinrichs et al., 2012).

One target of ROS- and redox-mediated retrograde signals is the gene for stromal ascorbate peroxidase (sAPX), which is involved in the chloroplast antioxidant defence system (Oelze et al., 2012). Here, it is shown that the transcription factor ANAC089 is localized to the trans-Golgi network and the ER, and is released upon treatment with reducing agents and targeted to the nucleus to bind to the *sAPX* promoter. Thus, ANAC089 might function in a negative retrograde signaling loop, lowering *sAPX* expression if the cell encounters highly reducing conditions (Klein et al., 2012).

## Progress in redox-associated signaling

Redox signals from photosynthesis itself report functional disturbances in photosynthesis. Their relation to the environment, potential transduction pathways to the nucleus and their impact on NGE are reviewed by Pfalz et al. (2012). Based on a review of the literature and of publicly available expression data, Tikkanen et al. (2012) postulate that changes in redox homeostasis at the thylakoid membrane, governed by thylakoid protein kinase STN7, reprogram the entire regulatory network in the cell. This idea underlines the need to consider retrograde signaling as part of a broader cellular network, instead of viewing it as a set of separate pathways (Estavillo et al., 2012). Hüner et al. (2012) focus on specific examples of acclimation to excess irradiance and temperature to illustrate how excitation pressure sensed within the chloroplast governs both local and remote molecular events that affect phenotypic plasticity. Further complexity in the major signaling pathways arises from their multiple interdependencies. Indeed, a genetic approach has revealed that signals related to the thylakoid redox state are also fed into the OGE-dependent retrograde pathway to modulate NGE and adjust the abundance of chloroplast proteins (Tadini et al., 2012). Lepistö et al. (2012) discuss evidence showing interaction of retrograde with light signaling pathways. Moreover, they present a hypothesis which proposes that heterogeneity in the plastid population can give rise to elaboration of distinct retrograde signals, based on NGE analysis of a mutant containing both photosynthetically active and non-photosynthetic plastids in a single mesophyll cell.

A valuable tool for the dissection of redox-dependent retrograde signaling is the *immutans* mutant, in which a lack of plastid terminal oxidase (PTOX) causes the formation of white and green sectors. The report by Foudree et al. (2012) provides an update on PTOX, the mechanism of *immutans* variegation, and discusses findings pertaining to compensatory mechanisms in the mutant.

Remarkably, the cofactors that supply many of the major post-translational modifications are either central metabolites or redox-active compounds. Hartl and Finkemeier (2012) evaluate the potential of phosphorylation, lysine acetylation, and glutathionylation not only to regulate organellar processes by modifying metabolic enzymes, but also to influence NGE. The retrograde signal transduction network might also encompass the dynamic, redox-dependent formation of microcompartments, as proposed by Wojtera-Kwiczor et al. (2012). Their experiments suggest redox-dependent binding of the glycolytic enzymes cytosolic glyceraldehyde-3-phosphate dehydrogenase and aldolase to the outer mitochondrial membrane and also to F-actin.

## Progress in tetrapyrrole-associated signaling

How exactly tetrapyrrole biosynthesis is associated with retrograde signaling and alters NGE remains elusive. Because of its central role as the rate-limiting step in tetrapyrrole biosynthesis, Czarnecki et al. (2012) chose to focus on the role of 5-aminolevulinic acid (ALA) biosynthesis in modulating NGE. These authors have investigated (1) ALA synthesis in the *genomes uncoupled* (*gun*) mutants *gun1*-*gun4* that show uncoupling of NGE from the physiological state of chloroplasts and assessed (2) the impact of post-translationally down-regulated ALA synthesis in gabaculine-treated seedlings and the *gun4-1* mutant by global transcriptome analysis. Another model postulates that a specific heme pool generated by flux through ferrochelatase I serves as a signal source (Woodson et al., 2011). Terry and Smith (2013) propose that this heme-related signal is the primary positive signal during chloroplast biogenesis. In addition to this positive signal, aberrant chloroplast development may produce a negative signal due to accumulation of unbound chlorophyll bio-synthesis intermediates, such as photo-excited Mg-porphyrins, which generate ^1^O_2_. Accordingly, the tetrapyrrole pathway may provide both positive and inhibitory signals to control NGE.

## Mitochondria and chloroplast crosstalk

Yet another level of complexity in organelle-nucleus crosstalk exists in photosynthetic eukaryotes because of the additional interactions between mitochondria and chloroplasts. Current evidence suggesting that the transcription factor ABA INSENSITIVE4 (ABI4) is important for both chloroplast and mitochondrial retrograde signaling pathways is presented by León et al. (2012). Van Aken and Whelan (2012) have analysed 27 microarray data sets relating to perturbations of chloroplast and mitochondrial function. Their results indicate that WRKY transcription factors play an important role in coordinating signaling from both organelles. Furthermore, new marker genes have been identified that respond specifically to mitochondrial and/or chloroplast dysfunction.

## Dual localized proteins as communicators

Proteins that are found both in the energy-producing organelles and the nucleus are excellent candidates for communicating information between these compartments. WHIRLY1 is such a protein, since it can be translocated from chloroplasts to the nucleus (Isemer et al., 2012b). Here, Isemer et al. (2012a) show that plastid-located WHIRLY1 enhances the responsiveness of seeds toward ABA even when ABA is supplied exogenously.

Duchêne and Giegé (2012) review the identified instances of proteins that are found both in mitochondria and the nucleus, most of which seem to be related to gene expression regulation, and propose that some of them might act as retrograde signaling proteins for mitochondrial biogenesis. Further proteins involved in OGE and, presumably, NGE are proteins of the mitochondrial Transcription tERmination Factor (mTERF) family. Thus, mTERFs represent ideal candidates for coordination of the expression of organelle and nuclear genomes (Kleine, 2012).

## Outlook

Many research efforts have focused on dissecting retrograde signaling pathways using biochemical and genetic approaches. In addition, metabolomics and systems biology have great potential to promote hypothesis generation and help dissect signaling networks in an unbiased fashion. Here, Caldana et al. (2012) outline and discuss recent advances in elucidating retrograde signaling molecules and pathways, with an emphasis on metabolomics- and systems biology-driven approaches.

The concept of retrograde signaling posits that signaling factors are generated in the organelles, are exported from the organelles, traverse the cytosol, and act in the nucleus. This notion is critically discussed by Leister (2012), highlighting the alternative scenario of a signaling factor that is actively exported from the organelle, such that NGE could be altered without changing the total concentration of the signaling molecule. Furthermore, the possibility must be considered that the signaling molecules generated in the organelle and the factors that trigger NGE are not necessarily identical. Finally, Estavillo et al. (2012) outline missing links or future areas of research that need to be addressed if we wish to gain a comprehensive understanding of plant intracellular signaling networks.
